# Screening for Specific Language Impairment in Preschool Children: Evaluating a Screening Procedure Including the Token Test

**DOI:** 10.1007/s10936-017-9493-z

**Published:** 2017-05-04

**Authors:** Ulrike Willinger, Michaela Schmoeger, Matthias Deckert, Brigitte Eisenwort, Benjamin Loader, Annemarie Hofmair, Eduard Auff

**Affiliations:** 1 Department of Neurology, Medical University of Vienna, Waehringer Guertel 18-20, 1090 Vienna, Austria; 2 Department of Pediatrics and Adolescent Medicine, Medical University of Vienna, Waehringer Guertel 18-20, 1090 Vienna, Austria; 3 Department of Otorhinolaryngology, Medical University of Vienna, Waehringer Guertel 18-20, 1090 Vienna, Austria

**Keywords:** Specific language impairment, Preschool children, Token Test, Language development, Intelligence

## Abstract

Specific language impairment (SLI) comprises impairments in receptive and/or expressive language. Aim of this study was to evaluate a screening for SLI. 61 children with SLI (SLI-children, age-range 4–6 years) and 61 matched typically developing controls were tested for receptive language ability (Token Test—TT) and for intelligence (Wechsler Preschool-and-Primary-Scale-of-Intelligence—WPPSI). Group differences were analyzed using *t* tests, as well as direct and stepwise discriminant analyses. The predictive value of the WPPSI with respect to TT performance was analyzed using regression analyses. SLI-children performed significantly worse on both TT and WPPSI ($p \le .0001$). The TT alone yielded an overall classification rate of 79%, the TT and the WPPSI together yielded an overall classification rate of 80%. TT performance was significantly predicted by verbal intelligence in SLI-children and nonverbal intelligence in controls whilst WPPSI subtest arithmetic was predictive in both groups. Without further research, the Token Test cannot be seen as a valid and sufficient tool for the screening of SLI in preschool children but rather as a tool for the assessment of more general intellectual capacities. SLI-children at this age already show impairments typically associated with SLI which indicates the necessity of early developmental support or training. Token Test performance is possibly an indicator for a more general developmental factor rather than an exclusive indicator for language difficulties.

## Introduction

Specific language impairment (SLI) is a neurodevelopmental disease that comprises impairments in receptive or/and expressive language (DSM-V, American Psychiatric Association 2013) in terms of “impaired comprehension and/or use of spoken, written and/or other symbol systems” (American Speech-Language-Hearing Association 2016). SLI is a common disorder in preschool children (prevalence rate of 7.4%, see Tomblin et al. 1997), appears more often in boys (Tomblin et al. 1997) and often persists into adolescence and adulthood (Gillam and Kamhi 2010; Clegg et al. 2005; Records et al. 1992; Stothard et al. 1998). SLI is often associated with behavioral problems (Willinger et al. 2003), impairments in spelling and reading (Willinger et al. 2001), executive functions (Henry et al. 2012), nonverbal and verbal intellectual capacity (Willinger and Eisenwort 1999), working memory (Dyck and Piek 2010), response inhibition (Dyck and Piek 2010) as well as motor problems (Noterdaeme et al. 2002). Furthermore, such language deficits are strongly associated with disorders and diseases like e.g. down syndrome, autism spectrum disorder, Williams syndrome or ADHD (Helland et al. 2012; Rice et al. 2005). Additionally, SLI is assumed to be a marker of increased vulnerability to develop a disorder in the schizophrenia spectrum (Mouridsen and Hauschild 2008). Imaging studies show that children with language impairments exhibit differences in brain structure, volume and functionality (Girbau-Massana et al. 2014). Furthermore, it could be shown that children with focal epilepsy are more likely to show a SLI (Parkinson 2002). The causes of SLI remain unclear; at least it could be shown that hippocampal abnormalities (Agostini et al. 2010) as well as genetic factors (Bartha-Doering et al. 2016; Kang and Drayna 2011) are involved whilst the heritability seems to be greater for more severe language impairments (Viding et al. 2004). Furthermore, children whose language disorders persist past the early childhood years show poorer psychosocial outcomes (Snowling et al. 2006). Considering these facts as well as the early onset of SLI, the high prevalence rate and the association between SLI and cognitive as well as behavioral problems, the necessity for an early screening for SLI is clearly given.

A method that seems to be suited for the early diagnostics of language impairments is the “Token Test” (DeRenzi and Vignolo 1962) which measures comprehension of verbal commands of increasing complexity (Strauss et al. 2006). The original version of the Token Test (DeRenzi and Vignolo 1962) was designed to assess comprehension of non-redundant commands in order to detect receptive disorders in aphasia patients. The Token Test allows for an assessment of linguistic capacities within a relatively brief period of time (Lass and Golden 1975) and shows high correlations with tasks measuring language reception as well as production (Cole and Fewell 1983; Gutbrod et al. 1985; Lass and Golden 1975). Evaluation studies could show that the Token Test successfully distinguishes between children with language impairments and non-impaired children, even in preschool age (see e.g. Cole and Fewell 1983; Geyer et al. 1978; Shelton et al. 1977). In this context, impairments on the Token Test were shown in children with closed head injury (Ewing-Cobbs et al. 1987), low birth weight (Hams et al. 1983), developmental aphasia (Tallal et al. 1985) developmental delay (Cole and Fewell 1983), autism (Minshew et al. 1995) and dyslexia (Slaghuis et al. 1993), for a review see Strauss et al. (2006). Furthermore, it could be shown that performance in the Token Test improves in childhood and that about age 11 children reach adult scores (Rich 1993; Strauss et al. 2006). The Token Test is also potentially suited to assess more general and complex verbal communication skills or even some general developmental factor (DiSimoni and Mucha 1982; Geyer et al. 1978; Remschmidt et al. 1977). In this context, it was shown that Token Test performance shows a moderate correlation with intelligence scores in brain-damaged subjects (Coupar 1976) but is associated with general cognitive ability (McNeil 1983; Riedel and Studdert-Kennedy 1985).

Despite the fact that tests for an early screening for language impairments exist and such screening tools are recommended as part of routine developmental surveillance, at least in the USA only approx. 45–70% of the children are screened for language deficits by their health care provider (for an extensive report see Siu 2015). Furthermore, Gillam and Kamhi (2010) note that there is the possibility that clinicians might not execute certain tasks in order to diagnose SLI (like e.g. measures of nonverbal intelligence) because they feel that those assessments do not fall within their scope of practice. Considering these points, the purpose of the current study was to investigate whether a short version (50 items) of the Token Test (Orgass 1982) is suitable for a simple and short screening for SLI in young childhood.

Strauss et al. (2006, for a review) name a number of advantages with respect to the Token Test, namely its cost-effectiveness in terms of material, time, and expertise required in administration and scoring; portability; sound discriminative validity; reasonable reliability and that the short versions of the Token Test show discriminant validity that is similar to that of the original version (for the last point see e.g. Taylor 1998). The authors of the current study name further advantages like clear instructions as well as its game-like character involving colorful objects (small and large circles and rectangles) that potentially maintains children’s motivation. Furthermore, it was shown that gender as well as ethnicity seem not to affect performance on the Token Test (see e.g. Peña-Casanova et al. 2009; Ripich et al. 1997). Strauss et al. (2006) also name disadvantages of the Token Test, namely that it relies on a limited stimulus array and that multiple reasons besides comprehension deficits might explain why patients perform poorly on the task.

Due to the Token Test’s advantageous properties, which comply with the requirements for the feasibility of screenings for speech and language disorders in young children (see e.g. Siu 2015), it is potentially highly feasible for the screening for SLI even in younger children. In order to strengthen such assumptions, the extent of the Token Test’s diagnostic value needs to be evaluated. In order to investigate whether also in preschool age Token Test performance is associated with general cognitive ability, the relationship between Token Test performance and intellectual capacities in preschool children with SLI and in preschool children without SLI was analyzed.

## Methods

### Subjects

*SLI-children *A sample of 61 preschool children was recruited at the Department of Otorhinolaryngology (Medical University of   Vienna) and comprised 16 girls (26%) and 45 boys (74%) with an expected majority of boys (Chi-squared—$\chi ^{2} (1, N = 61) = 13.79, p \le .0001$) and a mean age of 4.9 years (*SD* = 8.04 months, range: 3.9-6.2 years). SLI was diagnosed according to DSM-V (American Psychiatric Association 2013) as all children showed early occurring (Criterion C) and persistent language difficulties due to deficits in comprehension or production (Criterion A). The children showed no neurological, sensory, intellectual, or speech-motor deficits (Criterion D) but language performances that were substantially and quantifiably (1 SD) below those expected for age (Criterion B). Language abilities were assessed using the “Peabody Picture Vocabulary Test” (Dunn 1965), the “Heidelberg Evaluation of Language Development” (Grimm and Schoeler 1990) and the “Active Vocabulary Test for 3- to 6-year-old Children” (Kiese and Kozielski 1996). All children were native German speakers and all tests were conducted in German language. Prior to participation, the investigators made sure that all SLI-children comprehended the used terms of the Token Test objects (forms and colors); no child was excluded in this selection process. 59 children had been in kindergarten (98%) whilst two children had not been in a day care program (2%). Whilst 42 did not receive any treatment before participating in this study (69%), 15 had received speech therapy (24%), three received occupational therapy (5%), and one child received physical therapy (2%). Informed consent was obtained from the parents or legal guardian of each individual participant included in the study. The study protocol was approved by the ethics committee of the Medical University of Vienna.

*Control sample *The control sample consisted of 61 typically developing children without SLI (TD-children), matched to the SLI sample with respect to gender, age, and native language (only German). Prior to participation, the investigators made sure that all TD-children comprehended the used terms of the Token Test objects (forms and colors); no child was excluded in this selection process. No child had received any form of treatment prior to participating in this study whilst all of them had been to kindergarten ($M = 17.7$ months, *SD* = 8.1). Informed consent was obtained from the parents or legal guardian of each individual participant included in the study. The study protocol was approved by the ethics committee of the Medical University of Vienna.

### Materials

*Token Test *In this study, a short version (50 items) of the Token Test (Orgass 1982) was used in order to screen for SLI. In a standardized order, children are consecutively shown small and large plastic objects being different in form (rectangles and circles) and color (green, white, yellow, red, and blue). While being presented with these objects, children are required to understand and execute certain instructions like e.g. “Touch the blue rectangle”. Throughout the five parts of the task the demands become increasingly difficult as the instructions become progressively longer and more complex like e.g. “Touch the white circle after taking away the yellow rectangle”. Responses are scored dichotomously (correct/incorrect) and all incorrect responses are summed yielding a Token Test total error score.

*Intelligence scores *Intelligence was assessed using the German version (Eggert 1975) of the “Wechsler Preschool and Primary Scale of Intelligence“ (WPPSI) (Wechsler 1967) containing eight subtests. A nonverbal scale (subtests: mazes, geometric design, block design) as well as a verbal scale (subtests: comprehension, vocabulary, information) were calculated. Additionally, the subtests “animal house” assessing visual attention, visual memory and fine motor coordination as well as the subtest “arithmetic” assessing math knowledge, systematic problem-solving ability and working memory in a verbal modality were conducted.

### Statistics

#### Hypothesis 1

There will be a significant difference between SLI-children and TD-children with respect to Token Test performance (total score) as well as WPPSI performance (verbal and nonverbal scales plus two additional subtests). These differences will be analyzed for significance by *t* tests.

#### Hypothesis 2

Token Test performance (total score) will significantly classify SLI-children as well as TD-children and yield acceptable classification rates (80% or greater, see e.g. Plante and Vance 1994). These classification rates will be analyzed using a stepwise discriminant analysis.

#### Hypothesis 3

Token Test performance (total score) and WPPSI performance (verbal and nonverbal scales plus two additional subtests) will significantly classify SLI-children as well as TD-children and yield acceptable classification rates (80% or greater, see e.g. Plante and Vance 1994). These classification rates will be analyzed using a direct discriminant analysis.

#### Hypothesis 4

Token Test performance (total score) will be significantly predicted by WPPSI performance (verbal and nonverbal scales plus two additional subtests) in SLI-children. Within the SLI sample a regression analysis will be conducted using the total score of the Token Test as predicted variable and the results of the four parts of the WPPSI as the predictive variables.

#### Hypothesis 5

Token Test performance (total score) will be significantly predicted by WPPSI performance (verbal and nonverbal scales plus two additional subtests) in TD-children. Within the control sample a regression analysis will be conducted using the total score of the Token Test as predicted variable and the results of the four parts of the WPPSI as the predictive variables.

The cut-off level for statistical significance was set at $p< .05$. Therefore, with respect to hypotheses one, two and three, a significance level lower .05 leads to the rejection of the null-hypotheses and to the confirmation of the alternative hypotheses, respectively. With respect to hypotheses four and five, only statistically significant predictive variables ($p < .05$) will be interpreted as positive results and therefore seen as meaningful predictors of Token Test performance and subsequently discussed. Furthermore, the explained variance ($r^{2}$) by these meaningful predictors will be discussed. Data handling and analyses were carried out using SPSS for Windows, Version 20.

## Results

### Hypothesis 1

Group comparisons between SLI-children and TD-children showed significant differences with respect to the total score of the Token Test ($T(120) = 10, p \le .0001$) with SLI-children showing significantly more errors than controls (see Table 1). Significant differences between SLI-children and TD-children were also found with respect to intellectual capacities, as SLI-children exhibit lower scores in all four parts of the WPPSI (see Table 1).

**Table 1:** Group differences between SLI-children and typically developing children (controls) with respect to the Token Test total error score and the WPPSI scores

Variables	SLI	Controls	T-value
Token Test total error score$^\mathrm{a}$	*25.9 (15.3)*	*5.7 (6.6)*	*10*.*0*
WPPSI—Verbal Intelligence	*28.2 (13.0)*	*45.9 (16.7)*	$-$ *6*.*0*
WPPSI—Nonverbal Intelligence	*21.0 (13.3)*	*30.4 (12.4)*	$-$ *5*.*4*
WPPSI—Arithmetic	*5.8 (4.0)*	*10.8 (2.7)*	$-$ *10*.*8*
WPPSI—Animal House	*16.0 (8.3)*	*23.2 (7.0)*	$-$ *5*.*3*

Values are presented as mean with standard deviation (SD)

Significant results ($p \le .0001$) are italicized

*WPPSI* Wechsler Preschool and Primary Scale of Intelligence, *SLI* Children with specific language impairment

$^\mathrm{a}$ Higher scores mean worse performance

### Hypothesis 2

Stepwise multivariate discriminant analysis with the Token Test total score and the four parts of the WPSSI showed a significant discriminant function (Canonical Correlation = .7, Wilk’s Lambda = .6, $\chi ^{2} (5, N = 122) = 68.1, p\le .0001$) by the Token Test total score. 89% of the TD-children and 69% of the SLI-children could be classified correctly by the Token Test total score alone, yielding an overall correct classification of 79%.

### Hypothesis 3

Direct multivariate discriminant analysis of the Token Test total score and the four scores of the WPPSI showed a significant discriminant function (Canonical Correlation = .7, Wilk’s Lambda = .5, $\chi ^{2} (5, N = 122) = 71.6, p \le .0001$). 92% of the TD-children and 68% of the SLI-children could be classified correctly by the Token Test total score and the four scores of the WPSSI, yielding an overall correct classification of 80% (see Table 2).

**Table 2:** Direct discriminant analyses between SLI-children and typically developing children (controls) with respect to the Token Test total error score and the WPPSI scores

Variables	Wilk’s Lambda	F-value
Token Test total error score	.*560*	*92.6*
WPPSI—Verbal intelligence	.*743*	*40.8*
WPPSI—Nonverbal intelligence	.*889*	*14.8*
WPPSI—Arithmetic	.*658*	*61.2*
WPPSI—Animal houses	.*809*	*27.9*

*WPPSI* Wechsler Preschool and Primary Scale of Intelligence, *SLI* Children with specific language impairment

Significant results ($p \le .0001$) are italicized

### Hypothesis 4 and 5

Multiple regression analyses, separately calculated for SLI-children and TD-children, showed that among the SLI-children 63% ($r^{2} = .63, F = 23.318, p\le .0001$) and among the TD-children 30% ($r^{2} = .30, F = 5.864, p = .001$) of the variance of the Token Test total score were significantly explained by intellectual capacities. In SLI-children the Token Test total score was significantly predicted by the WPPSI verbal and WPPSI arithmetic scores as lower scores in both constructs were associated with more incorrect responses in the Token Test (see Table 3). In TD-children the Token Test total score was significantly predicted by the WPPSI nonverbal scale and WPPSI arithmetic scores as lower scores in both constructs were associated with more incorrect responses in the Token Test (see Table 4).

**Table 3:** Regression analysis scores in SLI-children with the Token Test total error score as the predicted variable and the four WPPSI scores as predictive variables

Intellectual abilities	Beta	T-value	*p* value
WPPSI—Verbal scale	$-$.*236*	$-$ *3*.*222*	.*002*
WPPSI—Nonverbal scale	.046	.588	.558
WPPSI—Arithmetic	$-$.*573*	$-$ *6*.*251*	.*000*
WPPSI—Animal house	$-$.132	$-$1.733	.086

*WPPSI* Wechsler Preschool and Primary Scale of Intelligence, *SLI* Children with specific language impairment

Significant results are italicized

**Table 4:** Regression analysis scores in typically developing children (controls) with the Token Test total error score as the predicted variable and the four WPPSI scores as predictive variables

Intellectual abilities	Beta	T-value	*p* value
WPPSI—Verbal scale	$-$.052	$-$.426	.672
WPPSI—Nonverbal scale	$-$.*287*	$-$ *2*.*038*	.*046*
WPPSI—Arithmetic	$-$.*626*	$-$ *4*.*302*	.*000*
WPPSI—Animal house	.178	1.446	.154

*WPPSI* Wechsler Preschool and Primary Scale of Intelligence

Significant results are italicized

## Discussion

SLI is one of the most common childhood disorders and often persists into adolescence and adulthood. SLI is often associated with multiple cognitive, motor, and behavioral problems and can be seen as a marker of increased vulnerability to develop psychiatric disorders. Whilst screenings and assessments of SLI are available, symptoms of SLI are often underestimated or overlooked due to various reasons. The aim of the study was to investigate whether the Token Test is suitable for a simple and short screening for SLI in young childhood.

In the current study, a difference between preschool children with SLI and typically developing children that were matched by age, gender and native language could be found with respect to the Token Test total error score as SLI-children showed significantly more errors. The diagnostic value of the Token Test as a suitable screening for SLI was further examined by discriminant analyses yielding a classification rate of 79%. Messick (1989) as well as Plante and Vance (1994) point out that a selected test must be valid for the purpose for which it will be used and that the interpretation of the test results as well as its implications for action should be validated, rather than the test itself. In the case of language tests such an interpretation should regard the presence or absence of a language impairment (Plante and Vance 1994) which should be supported by evidence and theoretical aspects (AERA, APA, & NCME 1999). So far, the Token Test shows a sound criterion validity as it was shown to be associated with measures of language reception and production (Cole and Fewell 1983; Gutbrod et al. 1985; Lass and Golden 1975) as well as a sound discriminative validity (Strauss et al. 2006). Furthermore, the Token Test was shown to successfully distinguish between language impaired and non-impaired children, even in preschool age (see e.g. Cole and Fewell 1983; Geyer et al. 1978; Shelton et al. 1977). In this study, the Token Test yielded a classification rate of 79% with respect to the presence or absence of SLI in preschool children. In their review, Plante and Vance (1994) note that for preschool language tests classification rates of 80–89% can be considered as fair whilst unacceptably high rates of misidentifications occur at classification rates below 80%. Therefore, the authors of the current study recommend that without further research the Token Test cannot be seen as a valid and sufficient tool for the screening of SLI in preschool age, as it does not sufficiently fulfill the purpose to identify children with SLI (see Plante and Vance 1994). Furthermore, the content validity, which usually relies on expert ratings (Plante and Vance 1994; Sireci and Sukin 2013), has to be viewed critically as it has been argued that the five parts of the Token Test do not measure the same general language factor (for a review see Strauss et al. 2006). With respect to the Token Test’s construct validity it was shown that despite its association with other language tasks (see e.g. Cole and Fewell 1983) it was also shown to be associated with general cognitive ability (see e.g. Riedel and Studdert-Kennedy 1985) and thought to even tap some general developmental factor (see e.g. DiSimoni and Mucha 1982). Furthermore, Strauss et al. (2006) note that besides comprehension deficits multiple factors might influence task performance like e.g. short-term memory, working memory, or inhibition (for a review see Strauss et al. 2006; but also Peña-Casanova et al. 2009). Therefore, it can be questioned to what extent - with respect to SLI - the Token Test really is sensitive to the intended construct and insensitive to irrelevant constructs (see e.g. AERA, APA, & NCME 1985, 1999).

In the current study, a difference between SLI-children and typically developing children could be found with respect to verbal intelligence; nonverbal intelligence; visual attention, visual memory and fine motor coordination; as well as mathematical ability, systematic problem-solving ability and working memory in a verbal modality as SLI-children showed significantly worse results. These results indicate that SLI-children at the age of four already show impairments that are typically associated with SLI (Dyck and Piek 2010; Henry et al. 2012; Noterdaeme et al. 2002; Willinger et al. 2001, 2003; Willinger and Eisenwort 1999). These results further reinforce the necessity for an early screening for SLI. Further analyses showed that adding these four intelligence scores yielded a marginally higher but acceptable classification rate of 80%. Seeing that the Token Test alone showed nearly the same predictive value, future studies should investigate whether the Token Test, modified versions or single parts of the Token Test can be used within shorter and more reliable screening procedures, given its advantageous properties.

In this study, the Token Test performance could be explained by different intelligence scores yielding a high rate of explained variance within the control group (30% in TD-children) as well as a remarkable rate with respect to the SLI children (62%). In both SLI-children and healthy controls the Token Test performance was significantly predicted by the WPPSI arithmetic score, a result that is not surprising considering that both tasks require to receive verbal information, process it in the working memory and execute the demanded action. An association between the Token Test performance and verbal intelligence was shown for SLI-children. This result is in line with previous studies investigating this relationship (see e.g. Kitson et al. 1985) as Remschmidt et al. (1977) consider the Token Test to measure general verbal communication and language development. Considering the result that in TD-children the Token Test performance was significantly predicted by nonverbal intelligence (reflecting the organization of visual perception with respect to visual motor coordination, perceptual motor function, visual analysis, synthesis and constructions, planning ability and visuospatial functions), it can be argued that the Token Test performance is a possible indicator for a more general developmental factor in preschool children rather than an exclusive indicator for language comprehension. In this context, Kitson et al. (1985) could show an association between Token Test performance and WISC performance IQ. Supporting the hypothesis that the Token Test assesses more than general and complex verbal communication skills, it could be shown that for the age-span 7–19 positive correlations can be found between language skills, intellectual abilities and the volume of the superior temporal gyrus which is thought to play a central role in auditory processing (Bigler et al. 2007).

## Conclusion

In the current study, preschool children with specific language impairment (SLI) showed significantly more errors on the Token Test than typically developing children. Yielding a classification rate of 79% by the Token Test performance the authors of the current study recommend that without further research the Token Test cannot be seen as a valid and sufficient tool for the screening of SLI in children aged between four and six years (for an review see Plante and Vance 1994) but rather as a tool for the assessment of more general intellectual capacities. Furthermore, the results indicate that SLI-children at the age of four already show impairments that are typically associated with SLI, indicating the necessity of an early screening for SLI as well as early developmental support or training of SLI-children. The Token Test performance could be explained by nonverbal as well as verbal intelligence scores, suggesting that it can possibly be seen as an indicator for a more general developmental factor rather than an exclusive indicator for language comprehension difficulties.
